# Mangrove Bacterial Diversity and the Impact of Oil Contamination Revealed by Pyrosequencing: Bacterial Proxies for Oil Pollution

**DOI:** 10.1371/journal.pone.0016943

**Published:** 2011-03-02

**Authors:** Henrique Fragoso dos Santos, Juliano Carvalho Cury, Flávia Lima do Carmo, Adriana Lopes dos Santos, James Tiedje, Jan Dirk van Elsas, Alexandre Soares Rosado, Raquel Silva Peixoto

**Affiliations:** 1 Laboratory of Molecular Microbial Ecology, Departamento of General Microbiology, Institute of Microbiology Paulo de Góes, Federal University of Rio de Janeiro, Rio de Janeiro, Rio de Janeiro, Brazil; 2 Center for Microbial Ecology, Michigan State University, East Lansing, Michigan, United States of America; 3 Department of Microbial Ecology, University of Groningen, Groningen, The Netherlands; Charité, Campus Benjamin Franklin, Germany

## Abstract

**Background:**

Mangroves are transitional coastal ecosystems in tropical and sub-tropical regions and represent biologically important and productive ecosystems. Despite their great ecological and economic importance, mangroves are often situated in areas of high anthropogenic influence, being exposed to pollutants, such as those released by oil spills.

**Methodology/Principal Findings:**

A microcosm experiment was conducted, which simulated an oil spill in previously pristine mangrove sediment. The effect of the oil spill on the extant microbial community was studied using direct pyrosequencing. Extensive bacterial diversity was observed in the pristine mangrove sediment, even after oil contamination. The number of different OTUs only detected in contaminated samples was significantly higher than the number of OTUs only detected in non-contaminated samples. The phylum Proteobacteria, in particular the classes Gammaproteobacteria and Deltaproteobacteria, were prevalent before and after the simulated oil spill. On the other hand, the order Chromatiales and the genus *Haliea* decreased upon exposure to 2 and 5% oil, these are proposed as sensitive indicators of oil contamination. Three other genera, *Marinobacterium*, *Marinobacter* and *Cycloclasticus* increased their prevalence when confronted with oil. These groups are possible targets for the biomonitoring of the impact of oil in mangrove settings.

**Conclusions/Significance:**

We suggest the use of sequences of the selected genera as proxies for oil pollution, using qPCR assessments. The quantification of these genera in distinct mangrove systems in relation to the local oil levels would permit the evaluation of the level of perturbance of mangroves, being useful in field monitoring. Considering the importance of mangroves to many other environments and the susceptibility of such areas to oil spills this manuscript will be of broad interest.

## Introduction

Mangroves are transitional coastal ecosystems in tropical and sub-tropical regions and represent biologically important and productive ecosystems [1], [2]. Currently, more than one third of the human population on Earth lives in coastal areas and on small islands, and the long-term sustainability of these populations depends on the contributions of coastal ecosystems, including coastal protection and fishery [3]. Despite their great ecological and economic importance, mangroves are often situated in areas of high anthropogenic influence, being exposed to pollutants, such as those released by oil spills [4], [5].

In fact, oil has a major ecological impact in the marine and terrestrial systems it contaminates (e.g. the recent accidents in the Gulf of Mexico and China). Thus, efficient strategies to monitor oil in the environment must be developed, especially in mangroves ecosystems [6]–[8].

Microorganisms are fundamental for the maintenance of productivity, conservation and recovery of mangroves. They are directly involved in the transformation of nutrients, photosynthesis, nitrogen fixation, methanogenesis, phosphate solubility, sulfate reduction and production of other substances, including antibiotics and enzymes and are reservoirs of products of biotechnological interest as, for example, bacteria that produce bioemulsifiers [7]. The knowledge about the effect of the oil spill on the extant microbial community in mangroves can provide possible targets for the biomonitoring of the impact of oil in mangrove settings.

High-throughput sequencing using sequencing-by-synthesis technology (454 pyrosequencing) has been introduced into microbial ecology as a new approach which is capable of revealing the taxonomic diversity within extant microbial communities at high resolution [9]–[11]. Amplification of the ribosomal RNA gene prior to pyrosequencing is an approach that can be used to describe the bacterial communities present in environmental samples [9], [12], [13]. Such an approach is very useful to extend our knowledge about the microbial communities that abound in mangrove ecosystems, and to evaluate the effects of oil spills on these.

In this study, the bacterial community structure and diversity in pristine mangrove sediment was evaluated using a 16S rRNA multiplex 454 pyrosequencing approach. We also evaluated the prevalence of bacterial targets that demonstrated to be sensitive to, or stimulated by the presence of oil in mangrove sediment microcosms that received heavy fuel oil.

## Materials and Methods

### Ethics Statement

The Institute of Microbiology Paulo de Góes, the Fundação Carlos Chagas Filho de Amparo à Pesquisa do Estado do Rio de Janeiro (FAPERJ) and the National Council for Research and Development (CNPq) approved this study development.

### Sampling site, microcosms and DNA extraction

This study was performed in mangrove sediment microcosms consisting of non-transparent 288.5 cm^3^ PVC tubes (7.5×7 cm). Each microcosm received 350 g (dry weight; 195 cm^3^) of fresh sediment per liter from the “Restinga da Marambaia”, Rio de Janeiro, Brazil (23°3′27″ S, 43°33′58″ W). The sediment (mud) sample was composed of ten sub-samples collected in a single location in the intertidal zone (20 cm deep). The composite sample was kept in a polyethylene bag that was transported to the laboratory, where the microcosms were immediately mounted (about 3 hours after sampling). Contamination with MF380 heavy fuel oil was established using two levels [2% and 5% (v/w)]. The oil was mixed into the sediments to create homogeneous sediments that were shared among all contaminated microcosms. Duplicate microcosms were analyzed on different days [i.e. day zero (T0, before contamination with oil), day 23 (T23 0%, T23 2% and T23 5%) and day 66 (T66 2%)]. Every 2 days, 100 ml of distilled water was added to each microcosm to replace evaporated water. A thin layer of water was present on top of each microcosm for ∼10 hours each time. The 8 microcosms were incubated in a greenhouse at ambient temperature (between 28–33°C).

To assess the structure of the bacterial communities associated with the mangrove sediment, 0.5 g samples of each sediment were used for DNA extraction using the Fast DNA Spin Kit for soil (QBIOgene, Carlsbad, CA, USA) following the manufacturer's instructions. The DNA obtained was quantified using a Qubit fluorometer apparatus (Molecular Probes, Invitrogen Detection Technologies, Oregon, USA).

### Assessment of total petroleum hydrocarbon (TPH) levels in sediment

We used 7–10 replicate 10-g aliquots (approximately 5 g dwt) from each sample for extraction with a dichloromethane∶acetone mixture (1∶1) in a Soxhlet extractor. Prior to the extraction, 100 ng of the standard (p-terfenil-d14) was added to the sample to comprise the aromatic fraction. The volume of the raw extract was reduced in an evaporator with rotary flow of N_2_ to yield a volume of 1 ml. Separation of fractions was accomplished by chromatography using a glass column, loaded with silica/alumina.

The determination of TPH was performed in a Varian Gas Cromatographer (GC) (CP 3800 MS Saturn 2200) equipped with a J&W (P/N 123-1334) DB-624 capillary column (30 m×0.32 mm I.D., 1.8 ìm) according to EPA methods 8015 and 8030. Helium was used as the carrier gas at a flow rate of 35 cm/sec, measured at 35°C. The initial temperature of the oven was 35°C, with an increase of 15°C/min (35–170°C). The split injector was set at 1∶40, and the injector temperature was set at 250°C. The injected volume was 1 µl. In the MSD detector, the detector temperature of the transfer line (full scan) was set at 280°C

### Pyrosequencing

Partial 16S rRNA gene sequences were amplified from replicates of sediment samples using the coded-primer (tag) approach to multiplex pyrosequencing. PCR amplification of the V4 region of the 16S rRNA gene was performed using 8-bp bar-coded eubacterial primers 563F and 802R (http://wildpigeon.cme.msu.edu/pyro/help.jsp). PCR mixtures were established as described in [13]. Equimolar amplicon suspensions were combined and subjected to pyrosequencing using a Genome Sequencer FLX system (454 Life Sciences, Branford, CT) at the Michigan State University Genomics Technology Support Facility. Sequences were excluded from the analysis if the read length was less than 150 bp or if the primer sequences contained errors (about 13%). Raw sequences were processed through the Ribosomal Database Project (RDP) pyrosequencing pipeline (http://wildpigeon.cme.msu.edu/pyro/index.jsp). Qualified sequences were clustered into operational taxonomic units (OTUs) defined by a 3% distance level using complete-linkage clustering and these were assigned to phyla using the RDP-II classifier at a 50% confidence threshold [14]. The 24,490 sequences obtained in this study were uploaded and are available at the GenBank under accessions numbers HM602044–HM622061 and HQ457546–HQ462469. Sequences that could not be classified into phyla at this confidence level were excluded from subsequent phylum composition analyses (about 503 sequences).

### Statistical analyses

A total of 24,490 partial 16S rRNA sequences were obtained from the ten sediment samples. Multiple sequence alignments for each sample were constructed with ClustalX. Based on the alignments, a distance matrix was constructed using DNAdist from the PHYLIP 3.6 package [15] with the default parameters in the Jukes-Cantor model option [16]. These pairwise distances served as inputs for DOTUR [17] for clustering the sequences into OTUs. The clusters were made at a 3% dissimilarity cut off and served as the OTUs for generating predictive rarefaction models and for calculating the richness indices Ace and Chao1 [18] and Shannon's diversity index [19]. Analyses for the Venn diagram generation were performed using the MOTHUR v. 1.14.0 suite of programs [17].

## Results and Discussion

**Figure 1:**
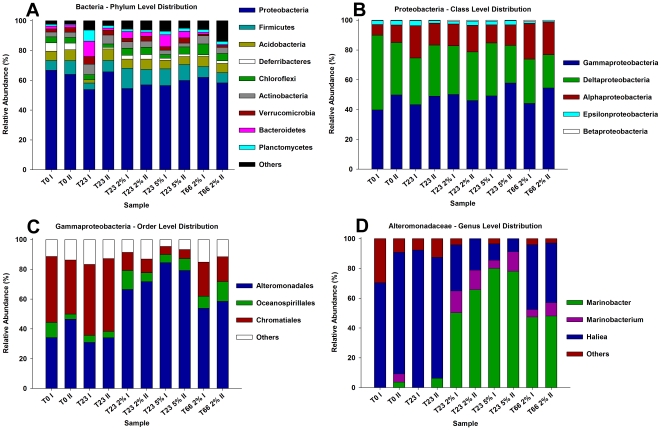
Composition of different phyla based on the classification of partial 16S rRNA sequences of bacteria from microcosm sediment using RDP-Classifier. Phyla (A); Proteobacteria classes (B), Gammaproteobacteria orders (C) and Alteromonadales genera (D). T0 and T23, without oil contamination in the beginning of the experiment and 23 days after oil contamination, respectively; T23 2% and T23 5%, 23 days after 2% or 5% oil contamination, respectively. Roman numerals distinguish the duplicate samples.

The analysis of Brazilian pristine mangrove sediment (before oil contamination) using pyrosequencing grouped the sequences obtained from the mangrove sediment samples into 22 different phyla. Of these, about 50–60% were related to those of Proteobacteria (Fig. 1A). This has been reported previously in mangroves using other molecular approaches [20], [21]. The dominant classes observed were Gammaproteobacteria and Deltaproteobacteria (Fig. 1B). The data obtained indicated a greater number of different phyla representatives than encountered in mangroves previously [20]–[23].

The Gammaproteobacteria were dominated by Chromatiaceae and Ectothiorhodospiraceae, both belonging to the order Chromatiales. Chromatiales is a group of anaerobic bacteria (known as Sulfur Bacteria), which is capable of photosynthesis by oxidizing hydrogen sulfide to sulfite and sulfate. The Deltaproteobacteria were dominated by the order Desulfobacterales, which is composed exclusively of (anaerobic) sulphate-reducing bacteria.

**Figure 2:**
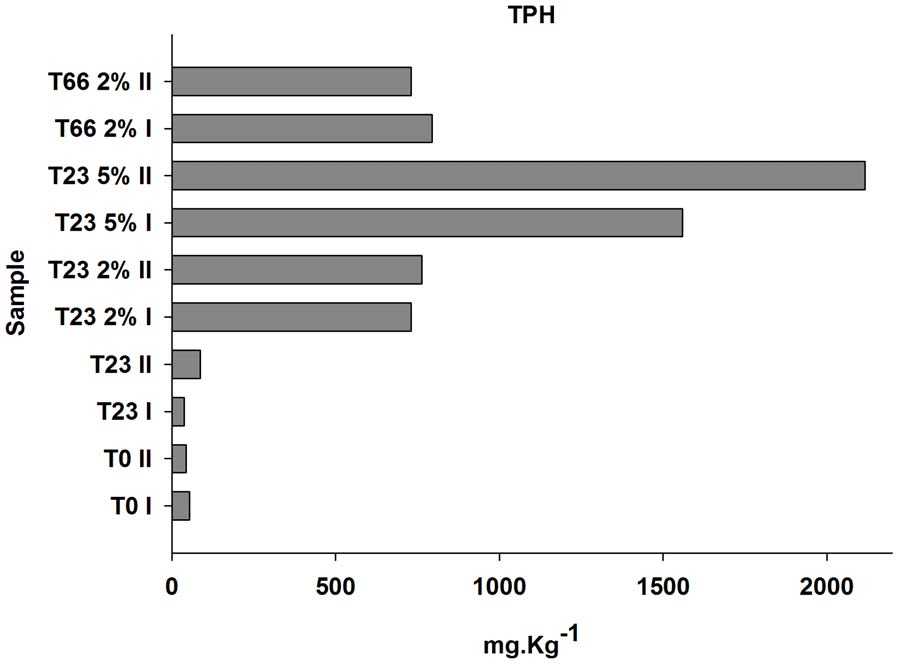
Total Petroleum Hydrocarbons (TPH) concentrations during experiment sampling. T0, T23 0, T23 2%, T23 5% and T66 2%: T0, without oil contamination; T23 0, 23 days after the beginning of the experiment without oil contamination; T23 2%, 23 days after 2% of oil contamination; T23 5%, 23 days after 2% of oil contamination; T66, 66 days after 2% of oil contamination. I and II indicates the duplicates.

**Figure 3:**
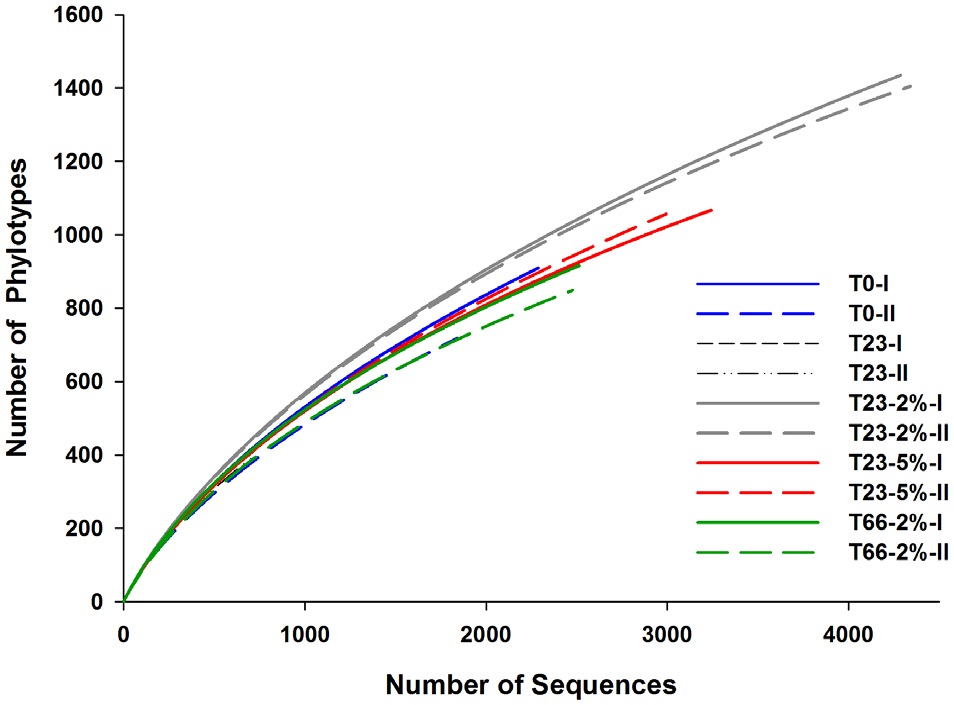
Rarefaction curves of partial sequences of 16S rDNA. The rarefaction curves from microcosm sediment samples, in duplicates, were calculated by DOTUR_003_. T0, T23 0, T23 2%, T23 5% and T66 2%: curves of 16S rDNA of each sampling. T0, without oil contamination; T23 0, 23 days after the beginning of the experiment without oil contamination; T23 2%, 23 days after 2% of oil contamination; T23 5%, 23 days after 2% of oil contamination; T66, 66 days after 2% of oil contamination. I and II indicates the duplicates.

There were clear differences in HTP levels from samples of different treatments (2 or 5% of oil concentration and non-contaminated samples), but no differences were observed considering sampling time [duplicates of T0 regarding duplicates of T23 0 (23 days after the beginning of the experiment without oil contamination) and T23 2% and T23 66% (23 and 66 days after 2% of oil contamination)] (Fig. 2). When the sediments were exposed to 2% of oil for 23 days, increases in species richness were detected using both the ACE and Chao 1 estimation methods (Table 1). Moreover, microbial diversity, as estimated by the Shannon index also increased (Table 1). The rarefaction curves indicated similar profiles of all samples (Fig. 3). We also normalized the data considering the same number of sequences to all samples (Table S1), and, despite the higher diversity of oiled samples were not so evident, the differences and similarities between samples were not significantly affected. In samples that had received 5% of oil, increases in bacterial richness and diversity also occurred, however to a lesser extent. This was probably caused by toxicity of the higher oil content.

**Table 1:** Estimated OTU richness, diversity indices and estimated sample coverage for 16S rRNA libraries of sediment of mangrove samples.

Biblioteca	NS^a^	OTUs^b^	Estimated OTU richness		Shannon^c^	ESC^d^
			ACE	Chao1		
T0 I	2265	910	2113 (1954; 2295)	2002 (1772; 2300)	6.22 (6.16; 6.27)	0.75
T0 II	1816	719	1695 (1497; 1944)	1582 (1387; 1846)	5.95 (5.89; 6.01)	0.76
T23 I	719	410	1015 (857; 1230)	960 (803; 1181)	5.70 (5.62; 5.78)	0.61
T23II	720	403	1050 (881; 1277)	987 (816; 1229)	5.66 (5.81; 5.74)	0.63
T23 2% I	4173	1435	2965 (2855; 3084)	2831 (2586; 3128)	6.57 (6.53; 6.61)	0.81
T23 2% II	4159	1405	2632 (2535; 2737)	2532 (2331; 2776)	6.54 (6.49; 6.58)	0.83
T23 5% I	3210	1067	2026 (1914; 2152)	1819 (1669; 2005)	6.26 (6.21; 6.31)	0.82
T23 5% II	2955	1057	2359 (2228; 2504)	2372 (2105; 2708)	6.22 (6.17; 6.28)	0.79
T66 2% I	2488	905	1833 (1726; 1953)	1827 (1626; 2084)	6.23 (6.19; 6.28)	0.79
T66 2% II	2436	837	1705 (1587; 1842)	1665 (1479; 1904)	5.97 (5.91; 6.03)	0.81
**Total**	**24941**					

a Number of sequences for each library.

b Calculated with DOTUR at the 3% distance level.

c Shannon diversity index calculated using DOTUR (3% distance).

d Estimated sample coverage: Cx = 1−(Nx/n), where Nx is the number of unique sequences and n is the total number of sequences.

Values in brackets are 95% confidence intervals as calculated by DOTUR.

The bacterial richness and diversity increases are probably due to the growth of diverse species on the degradable hydrocarbon fractions, or an increase of the degree of “structures” (niches) in the system. Hence, a limiting factor in the degradation of petroleum hydrocarbons in mangroves is not the intrinsic ability of its microbial community to degrade, which has already been described in several studies [7], [24], but possibly an imbalance in the ratio of C∶N∶P caused by the high carbon content of the oil. The oil may cause a rapid consumption of the nitrogen and phosphorus sources that are present, which are commonly scarce in mangrove sediment [22]. In turn, these changes in microbial diversity and structure, and nutrient ratio's, can cause shifts in other biological groups. For instance, in the same mangrove sediment, Santos and colleagues [8] detected a large decrease in the diversity and richness of microeukaryotes 23 days after contamination with 2% oil.

The relative abundances of phyla after the addition of the oil to the system did not change significantly (Fig. 1A). However, there were clear changes at finer levels of taxonomic resolution, after 23 and 66 days. While the class Gammaproteobacteria remained dominant before and after oil contamination (Fig. 1B), several orders within the Gammaproteobacteria showed major shifts after oil contamination. The orders Oceanospirillales and Alteromonadales increased significantly. Before contamination, the order Alteromonadales represented about 30–50% of the gammaproteobacterial sequences, but this level became 75% and 90% of the sequences 23 days after contamination with 2% and 5% oil, respectively, and 52 and 57% 66 days after contamination with 2% oil (Fig. 1C).

Because the orders Oceanospirillales and Alteromonadales showed major changes in the oil-treated samples, we also analyzed the bacterial distribution at the level of genera in these orders. In the Oceanospirillales, the genus that displayed the greatest change after contamination was *Alcanivorax* (from Latin, eater of alkanes). *Alcanivorax* species are alkane-degrading marine bacteria that propagate and become dominant in crude oil-containing seawater that is supplemented with nitrogen and phosphorus [25]. In our study, the 17 genera of Alteromonadales found, only *Marinobacterium* and *Marinobacter* showed significant increases after the addition of oil (Fig. 1D). Before contamination, the genus *Marinobacterium* and *Marinobacter* represented about 1% of the Alteromonadales sequences, but this level became about 45–50% 23 and 66 days after 2% of oil contamination and 70 to almost 80% after 23 days of 5% of oil contamination. The genera *Marinobacter* and *Marinobacterium* have already been described in several studies as being present in areas contaminated with oil [26]–[28], including mangroves [29]. They may be potential proxies for the biomonitoring of petroleum hydrocarbons in this ecosystem.

Other genera that showed significant increases after oil contamination and that can be proposed as bioindicators of oil contamination were *Clostridium* and *Fusibacter* (both belonging to sequences of the phylum Firmicutes). The genus *Haliea*, which represented about 90% of the sequences of Alteromonadales, only represented 30% and 10% of these sequences 23 days after addition of 2% and 5% oil, respectively, and 45% 66 days after addition of 2% oil. This indicated that these organisms are sensitive to oil. The order Chromatiales was also highly affected by the presence of oil. Representatives of this order constituted more than 50% of the gammaproteobacterial sequences before oil contamination, but this decreased to about 15% and 5% 23 days after addition of 2% and 5% oil, respectively, and about 20% 66 days after addition of 2% oil. The genus *Haliea* was recently described, isolated from marine coastal area [30], as well as the other two *Haliea* species described until this moment were also isolated from the marine environment [31], [32].

**Figure 4:**
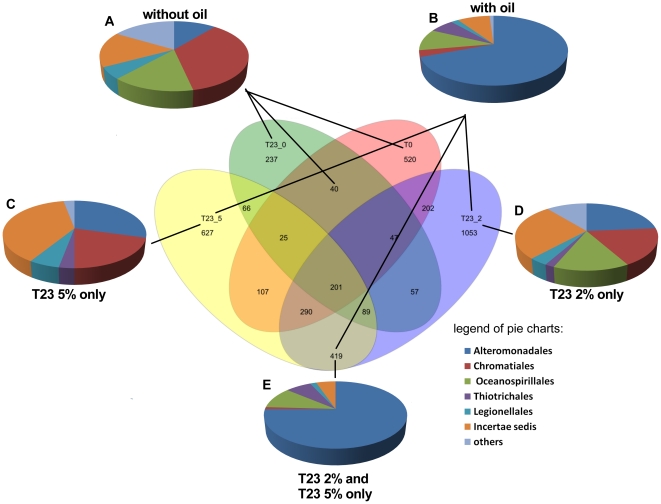
Venn Diagram evaluating the concentration of oil contamination. In the center of the figure, the Venn diagram is showing unique and sharing OTUs (97%) in each microcosm sample. The order designations of the sequences related to the unique OTUs were determined using the RDP Classifier tool. (A), Unique sequences of samples without oil contamination; (B), Unique sequences of samples contaminated with oil; (C), Unique sequences of samples with 5% of oil contamination; (D), Unique sequences of samples with 2% of oil contamination. ; (E), Unique sequences of samples with 2% and 5% of oil contamination.

**Figure 5:**
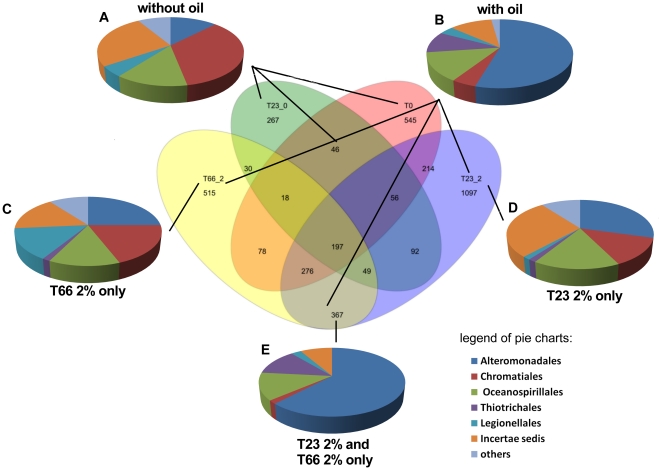
Venn Diagram evaluating the time of oil exposure. In the center of the figure, the Venn diagram is showing unique and sharing OTUs (97%) in each microcosm sample. The order designations of the sequences related to the unique OTUs were determined using the RDP Classifier tool. (A), Unique sequences of samples without oil contamination; (B), Unique sequences of samples contaminated with oil; (C), Unique sequences of samples 66 days after 5% of oil contamination; (D), Unique sequences of samples 23 after 2% of oil contamination; (E), Unique sequences of samples 23 and 66 days after 2% of oil contamination.

To evaluate only OTUs detected when oil was applied, Venn diagrams were constructed. The diagrams indicated specific and common OTUs of all treatments, showing a higher number of OTUs that were specific of oiled samples. While 2099 and 1979 OTUs were specific of samples contaminated with oil, when oil concentration (Fig. 4A–E) or time of exposition (Fig. 5A–E) were evaluated, respectively, 797 (Fig. 4) and 858 (Fig. 5) OTUs were specific of non-contaminated samples. These results are related to the higher microbial diversity in oiled samples observer in Table 1.

To identify more oil proxies using the Venn diagrams, we evaluated all sequences related to the specific oil OTUs and specific OTUs from samples without oil related to the dominant class, Gammaproteobacteria. Using only these sequences, it was possible to detect other orders that were affected by oil contamination that were not detectable in Figure 1c analysis. For instance, the Thiotrichales order was only detected in samples with oil even when oil time exposition or oil concentration were evaluated (Figs. 4A and 4B; Figs. 5A and 5B). The majority of Thiotricales OTUs were identified as belonging to *Cycloclasticus* genus. Bacteria belonging to this genus are described developing a primary role in the degradation of aromatic hydrocarbons released in a marine environment [33], [34], which could justify this predominance after oil contamination.

The Chromatiales order was dominant before oil contamination and decreased significantly after oil contamination, while Alteromonadales increased and became dominant after oil contamination (Figs. 4A–E; Figs. 5A–E) as also demonstrated when all sequences were evaluated (Fig. 1C). Although no significant differences were observed between T23 2% and T66 2% samples (Fig. 5C–D), as well as observed to the TPH levels (Fig. 2) there were clear differences related to oil concentrations. While the Oceanospirillales was an important order in samples exposed to 2% of oil contamination, this same order was not detected in samples exposed to 5% of oil contamination.

As an example of the application of our data, we suggest the use of sequences of the genera *Marinobacter*, *Marinobacterium*, *Cycloclasticus*, and *Haliea* as proxies for oil pollution, using qPCR assessments. The quantification of these genera in distinct mangrove systems in relation to the local oil levels would permit the evaluation of the level of perturbance of mangroves. Hence, our approach might be useful in field monitoring. However, further studies need to focus on the robustness of these proxies, applying isolation and microscopic techniques to monitoring these organisms in the face of oil contamination.

In mangrove sediments, the dominant electron acceptor in anaerobic biodegradation is sulfate [35]. We found a large number of sulfate reducing bacteria, both before and after oil contamination. Specific strategies of biostimulation in environments such mangroves could be obviously indicated to be applied *in situ*, by the addition of soluble electron acceptors, to assist in the removal of contaminants.

The survey of the indigenous microbial communities of the studied mangrove sediment and the possible impacts of oil on these is extremely important for the further monitoring of the biological quality of these. In the current study, the sediment was sampled in a mangrove area located in Sepetiba Bay, Rio de Janeiro, Brazil, where the Itaguaí Port is expanding to accommodate larger ships. This makes this preserved marine environment highly susceptible to an ecological disaster caused by an oil spill.

## Supporting Information

Table S1 **Estimation of OTU richness, diversity indices and estimated sample coverage for 16S rRNA libraries of sediment of mangrove samples. The number of sequences of each sample was normalized to 700.** a. Number of sequences for each library. B. Calculated with DOTUR at the 3% distance level. C. Shannon diversity index calculated using DOTUR (3% distance) d. Estimated sample coverage: Cx = 1−(Nx/n), where Nx is the number of unique sequences and n is the total number of sequences. Values in brackets are 95% confidence intervals as calculated by DOTUR.
(DOC)
