# Supplementary material for: Mangrove Bacterial Diversity and the Impact of Oil Contamination Revealed by Pyrosequencing: Bacterial Proxies for Oil Pollution
Source: PLoS One. 2011 Mar 2;6(3):e16943. doi: 10.1371/journal.pone.0016943 (PMC3047533; doi:10.1371/journal.pone.0016943)
Supplement: Table S1 — Estimation of OTU richness, diversity indices and estimated sample coverage for 16S rRNA libraries of sediment of mangrove samples. The number of sequences of each sample was normalized to 700. a. Number of sequences for each library. B. Calculated with DOTUR at the 3% distance level. C. Shannon diversity index calculated using DOTUR (3% distance) d. Estimated sample coverage: Cx = 1−(Nx/n), where Nx is the number of unique sequences and n is the total number of sequences. Values in brackets are 95% confidence intervals as calculated by DOTUR. (DOC) [file pone.0016943.s001.doc]

| Biblioteca | NS^a^ | | OTUs^b^ | Estimated OTU richness | | | Shannon^c^ | ESC^d^ |
| --- | --- | --- | --- | --- | --- | --- | --- | --- |
|  |  | |  | ACE | Chao1 | |  |  |
|  | | | | | | | | |
| T0 I | | 719 | 405 | 1112 (932; 1353) | 1035 (850; 1296) | 5.71 (5.64; 5.78) | | 0.61 |
| T0 II | | 719 | 383 | 1092 (904; 1348) | 1012 (822; 1284) | 5.58 (5.51; 5.66) | | 0.63 |
| T23 I | | 719 | 408 | 1005 (848; 1217) | 950 (795; 1168) | 5.69 (5.62; 5.77) | | 0.61 |
| T23II | | 719 | 393 | 1025 (859; 1251) | 957 (790; 1193) | 5.63 (5.55; 5.71) | | 0.63 |
| T23 2% I | | 719 | 431 | 1289 (1077; 1570) | 1184 (967; 1487) | 5.82 (5.75; 5.88) | | 0.57 |
| T23 2% II | | 719 | 437 | 1363 (1132; 1669) | 1180 (971; 1470) | 5.80 (5.73; 5.88) | | 0.56 |
| T23 5% I | | 719 | 397 | 1150 (955; 1413) | 931 (776; 1149) | 5.64 (5.56; 5.72) | | 0.62 |
| T23 5% II | | 719 | 385 | 1033 (862; 1267) | 801 (681; 970) | 5.57 (5.48; 5.65) | | 0.64 |
| T66 2% I | | 719 | 458 | 1011 (852; 1227) | 881 (739; 1083) | 5.65 (5.57; 5.72) | | 0.64 |
| T66 2% II | | 719 | 368 | 985 (819; 1213) | 937 (763; 1188) | 5.42 (5.33; 5.52) | | 0.65 |
